# Design characteristics of mass balance studies conducted for protein kinase inhibitors

**DOI:** 10.1007/s00280-026-04926-5

**Published:** 2026-07-13

**Authors:** Benthe Riechelman, Ramon Bolks, Thijs H. Oude Munnink, Jesse J. Swen, Frank G. A. Jansman, Frank Klont

**Affiliations:** 1 Unit of PharmacoTherapy, Epidemiology and Economics, Groningen Research Institute of Pharmacy, University of Groningen, Antonius Deusinglaan 1, Groningen, 9713 AV The Netherlands; 2 Stichting Beoordeling Ethiek Biomedisch Onderzoek (BEBO), Weiersstraat 1C, Assen, 9401 ET The Netherlands; 3 Department of Clinical Pharmacy and Pharmacology, University Medical Center Groningen, University of Groningen, Hanzeplein 1, Groningen, 9700 RB The Netherlands; 4 Department of Clinical Pharmacy & Toxicology, Leiden University Medical Center, Albinusdreef 2, Leiden, 2333 ZA The Netherlands; 5 Department of Clinical Pharmacy, Deventer Hospital, Nico Bolkesteinlaan 75, Deventer, 7416 SE The Netherlands

**Keywords:** Cancer, Drug metabolism, Pharmacokinetics, Protein kinase inhibitors

## Abstract

**Purpose:**

Protein kinase inhibitor (PKI) therapy increasingly relies on personalized approaches such as therapeutic drug monitoring and pharmacogenetic testing. Understanding PKI metabolism is essential for these methods to be effective, notably for determining the substance to be quantified and the enzyme for which the genotypic metabolizer status needs to be predicted. For most drugs, however, human in vivo metabolite profiles are derived from the mass balance studies conducted by drug developers, typically in small and homogeneous populations.

**Methods:**

We studied mass balance study characteristics for PKIs, assessing number of participants, sex, and health state, as well as dosing regimen, study location, study duration, and consideration of genetic variation in metabolic enzymes and transporters. Data were primarily extracted from European Public Assessment Reports, supplemented with information from the Drugs@FDA database, ClinicalTrials.gov, and peer-reviewed mass balance study publications.

**Results:**

Mass balance studies were conducted for 68 out of the 69 PKIs listed as “authorized” human drugs in the European Medicines Agency database on July 7, 2025 (updated April 18, 2026; +3 PKIs). The studies included 2–12 participants (median = 6), with 90% enrolling only healthy volunteers, 88% enrolling only males, 97% administering a single dose, and four studies mentioning consideration of genetic variation in metabolic enzymes during participant inclusion.

**Conclusion:**

PKI mass balance studies exhibit similar, rather homogeneous design characteristics, potentially limiting the generalizability of their insights into drug metabolism across diverse patient populations. Still, most align with recent FDA guidelines on mass balance studies, despite being issued after study conduct.

**Supplementary Information:**

The online version contains supplementary material available at 10.1007/s00280-026-04926-5.

## Introduction

Protein kinase inhibitors (PKIs) represent an important and emerging class of drugs, which are crucial for reducing the activity of kinases involved in cell proliferation and survival [1]. A large group of PKIs is particularly relevant in the context of personalized medicine, notably in oncology. For these drugs, therapy selection often depends on the presence of genetic mutations in specific receptors or signalling proteins, which will determine whether treatment is likely to achieve the desired effect or not [2]. In addition to this pharmacodynamic perspective on personalized medicine, PKIs are also of interest from a pharmacokinetic (PK) point of view. In particular, PKI therapy may be further personalized, for example through therapeutic drug monitoring (TDM), pharmacogenetic testing of metabolic enzymes, and PK modelling approaches [3–5].

Effective application of these personalization approaches requires detailed knowledge of whether and which PKI metabolites are formed in humans, as is usually elucidated during a radiolabelled mass balance study [6]. Such a phase I study is, however, often conducted in a limited number of participants, typically being healthy, male volunteers. In fact, a study assessing all drugs approved between 2014 and 2018 [7] indicated that out of the 104 mass balance studies analysed, 90% of the studies enrolled healthy individuals only, and only males were eligible for enrolment in 86% of the studies. In 66% of these studies, at least 6 individuals were included, while in 13%, fewer than six evaluable participants were reported. Hence, these populations may not be fully representative of the eventual users of these drugs, by insufficiently capturing the variability of pharmacokinetic processes in actual patient populations. Admittedly, various other clinical study types may provide insight into metabolite exposure across different population characteristics (e.g., sex, age, disease state, pharmacogenetic status); however, a full profile and quantification of drug metabolites is generally only obtained in vivo in humans through mass balance studies. Consequently, when potentially incomplete metabolite profiles are established, for example, in small, homogeneous populations, any resulting bias may persist despite subsequent evaluation of metabolite exposures in more diverse clinical settings. In this regard, recent real-world drug metabolism and excretion studies repeatedly demonstrate that drug metabolism and excretion in clinical practice can deviate from expectations based on preclinical or clinical studies [8, 9], which may potentially also apply to PKIs.

In this study, we aimed to systematically assess how mass balance studies have been performed for PKIs. Specifically, we examined study population characteristics, including sample size, sex distribution, and whether participants were healthy volunteers or patients, as well as dose regimen, study location, and study duration (taking into account the mean/median total recovery of the administered radioactive dose, which often determines duration). These parameters were evaluated in the context of the recently issued United States Food and Drug Administration (FDA) guidance on mass balance studies, which provides recommendations on study design aspects such as sample size, participant selection, dosing regimen, and recovery of radioactivity [10]. Moreover, the guidance recommends that variability in pharmacokinetics and relevant genetic polymorphisms should be considered when determining the number of participants for enrolment [10]. Therefore, we also assessed the extent to which pharmacogenetic factors were considered.

## Materials and methods

### Drug selection

This study focused on PKIs approved for therapeutic use in humans and listed in the European Medicines Agency (EMA) database, downloaded from https://www.ema.europa.eu/en/documents/report/medicines-output-medicines-report_en.xlsx on July 7, 2025 (with an updated search conducted on April 18, 2026). Drugs were selected based on the Anatomical Therapeutic Chemical (ATC) classification system, including only “authorized” drugs with the ATC codes L01E (“Protein kinase inhibitors”) and L01XE (“Protein kinase inhibitors”). When a drug was registered for two indications, the oldest indication was included as the respective marketing authorization documents typically contain more pharmacokinetic data.

### Data sources

For each included drug, the initial ‘European Public Assessment Report’ (EPAR) was retrieved from the EMA website (listed under “initial marketing authorization documents”) in July and August 2025. Matching documents, including ‘Clinical Pharmacology and Biopharmaceutics Reviews’, ‘Multidisciplinary Reviews’, and/or ‘Integrated Reviews’ were downloaded from the Drugs@FDA database of the FDA in July and August 2025. Additionally, mass balance study publications were searched for via PubMed between July and October 2025. Further clinical trial information was obtained from ClinicalTrials.gov between September and October 2025, and, if necessary, direct email contact with the manufacturers was established between August and October 2025 in case of difficulties or a lack of key information (not undertaken for the updated search). Lastly, data extraction primarily relied on the EPARs and was supplemented with available information from the peer-reviewed mass balance articles, FDA review documents, and ClinicalTrials.gov (for study location data only).

### Data extraction

The extracted data included number of participants, sex distribution, health status (patient/healthy volunteer), dosing regimen (single/multiple doses), study location, total recovery of radioactivity, and duration of sampling. When a peer-reviewed study publication was available, mentions of genetic variation in metabolic enzymes and transporters relevant for participant inclusion were searched in the Materials and Methods sections. Lastly, the primary/predominant drug-metabolizing enzyme was extracted from the EPAR.

## Results

### Overview of included protein kinase inhibitors

A total of 69 authorized PKIs for human use were retrieved from the EMA database on July 7, 2025, and an updated search conducted on April 18, 2026 identified three additional PKIs. Mass balance studies had been conducted for 71 of them. Only in the case of ripretinib, no mass balance study was conducted, which was reported to be due to drug formulation issues. At the time of conducting this study (between July and October 2025, with an updated search conducted in April 2026), published mass balance articles could be found for 48 PKIs, whereas studies of 6 PKIs were published as conference abstracts and one was published as a supplementary protocol to a “*registrational phase 1–2 trial*” publication. For 16 PKIs, no study publications were found, after which the corresponding drug developers were contacted to request further information (initial search only). In 12 cases, the lack of published information was confirmed, while no response was given for the remaining 3 PKIs. An overview of all included PKIs and all extracted study information is provided in Appendix 1.

### Number of participants

**Fig. 1:**
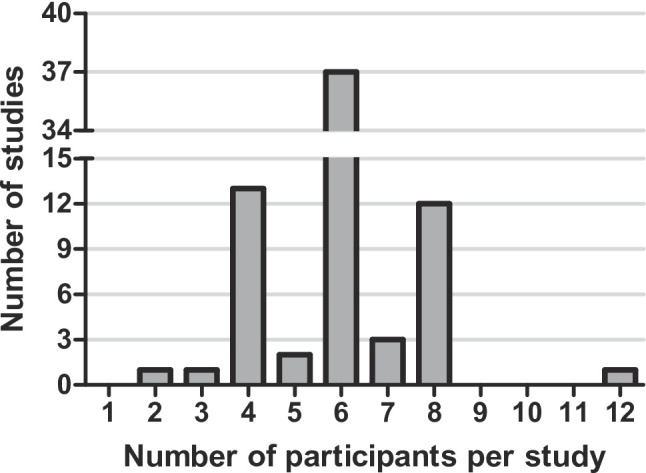
Histogram depicting the number of participants per PKI mass balance study. For the study with 12 participants, the distribution across the two study parts is unclear, with mass balance being assessed in only one part

Across the 70 PKIs for which the sample size could be assessed, the median number of evaluable participants per study was 6, with a minimum of 2 and a maximum of 12 (see Fig. 1). The actual maximum may, however, be lower, as it could not be retrieved how the 12 healthy male volunteers included in the larotrectinib trial were distributed over the two study parts, with mass balance only being assessed in one part. Apart from larotrectinib, the number of participants per study was six or higher in 52 studies (75%), which is consistent with the recent FDA guidelines (i.e., the first formal guidance document on human mass balance studies) stating that such studies “…*should include at least six evaluable volunteers who have completed the study procedures…*” [10]. Lastly, notable outliers in this context were the studies of trametinib and pazopanib, which included 2 and 3 male patients, respectively. The corresponding peer-reviewed publication for trametinib indicated early termination of the planned enrolment of 4 to 6 males (“*…due to low dose recovery and concern regarding revised estimates of radiation exposure…*”). In the case of pazopanib, no information was provided on potential enrolment issues or constraints.

### Sex distribution

Across the 69 PKIs for which sex distribution could be assessed, 61 studies (88%) were conducted exclusively in males, and 8 studies (12%) included both males and females. Among these, four studies (i.e., gilteritinib, lapatinib, lenvatinib, tucatinib) included equal numbers of males and females. The remaining four (i.e., acalabrutinib, dabrafenib, midostaurin, vemurafenib) had uneven distributions, with vemurafenib being the only PKI for which more females than males were evaluated in its mass balance study. Of the four PKIs not studied in patients (i.e., lapatinib, tucatinib, acalabrutinib, midostaurin), peer-reviewed study publications and/or ClinicalTrials.gov records indicated that females were required to be not of childbearing potential, with the exception of midostaurin, for which no further information on female inclusion could be retrieved.

### Participant type

The vast majority of mass balance studies were conducted in healthy volunteers, as only seven studies of 71 (10%) included patients. Specifically, dabrafenib was studied in patients with BRAF V600 mutation-positive tumors (1 female, 3 males), everolimus in renal transplant recipients (4 males), gilteritinib in patients with advanced solid tumors (3 females, 3 males; multiple dose regimen), lenvatinib in patients with advanced solid tumors or lymphomas (3 females, 3 males), pazopanib in patients with advanced cancer (3 males), trametinib in a patient with stage IV melanoma and a patient with hepatocellular carcinoma (2 males), and vemurafenib in patients with metastatic melanoma (4 females, 3 males; multiple dose regimen). Available peer-reviewed study publications substantiated the rationale for including patients in the cases of gilteritinib (“*…marked accumulation after multiple-dose administration (reflecting its intended use)…*”), trametinib (“.*in order to employ a higher dose of trametinib to enable metabolite analysis*”), and vemurafenib (“*[o]wing to occurrence of cutaneous squamous cell carcinoma (cuSCC) and secondary primary melanomas in other vemurafenib clinical studies…*”). These rationales were linked to safety considerations, consistent with the single reason for including patients described in recent FDA guidelines [10]. For dabrafenib and pazopanib, patients were allowed to transfer to rollover studies to receive continued treatment, (presumably) enrolling in studies registered in ClinicalTrials.gov (NCT01231594 and NCT00387205, respectively), which had safety as a primary outcome. In the case of lenvatinib, all participants automatically entered an extension phase to fulfil secondary objectives (i.e., assessment of safety and efficacy).

### Study location

**Fig. 2:**
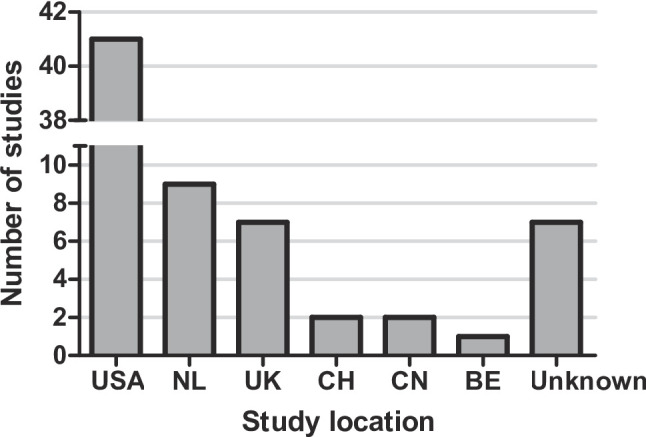
Histogram showing the distribution of study locations across PKI mass balance studies. Abbreviations: USA = United States of America, NL = The Netherlands, UK = United Kingdom, CH = Switzerland, BE = Belgium, CN = China

Study locations could be retrieved for 62 out of the 69 mass balance studies, which were all single-centre studies and were mainly conducted in North America and Europe (see Fig. 2). From these 62 studies, 41 (66%) were conducted in the United States, 9 (15%) in the Netherlands, 7 (11%) in the United Kingdom, 2 (3%) in Switzerland, 1 (2%) in Belgium, and 2 (3%) in China.

### Dosing regimen

Participants in 69 of 71 mass balance studies (97%) received a single dose of the investigational drug, with only two studies using multiple doses, both being conducted in patients. In the case of gilteritinib, patients received an unlabelled version of the drug for two weeks, and on day 15, a radiolabelled dose was administered, after which the unlabelled treatment was continued through day 47. The corresponding peer-reviewed study publication reported that “*[a]s gilteritinib has marked accumulation after multiple dose administration (reflecting its intended use)*,* the study was designed so that absorption*,* metabolism*,* and elimination could be evaluated at steady state*”. In the case of vemurafenib, the same protocol was used with the exception that the unlabelled treatment was continued until disease progression. In this case, the study publication reported that “*[v]emurafenib exhibits extensive accumulation at steady state*,* and characterization of single and multiple dose PK suggests that drug disposition is different at steady state than after a single dose*”, thereby substantiating the conduct of the mass balance study at steady-state. Lastly, both rationales for employing multiple dose designs are in line with the recent FDA guidance document, which provides two examples of relevant scenarios, namely time-dependent pharmacokinetics and studies conducted in patients [10].

### Study duration and recovery of radioactivity

**Fig. 3:**
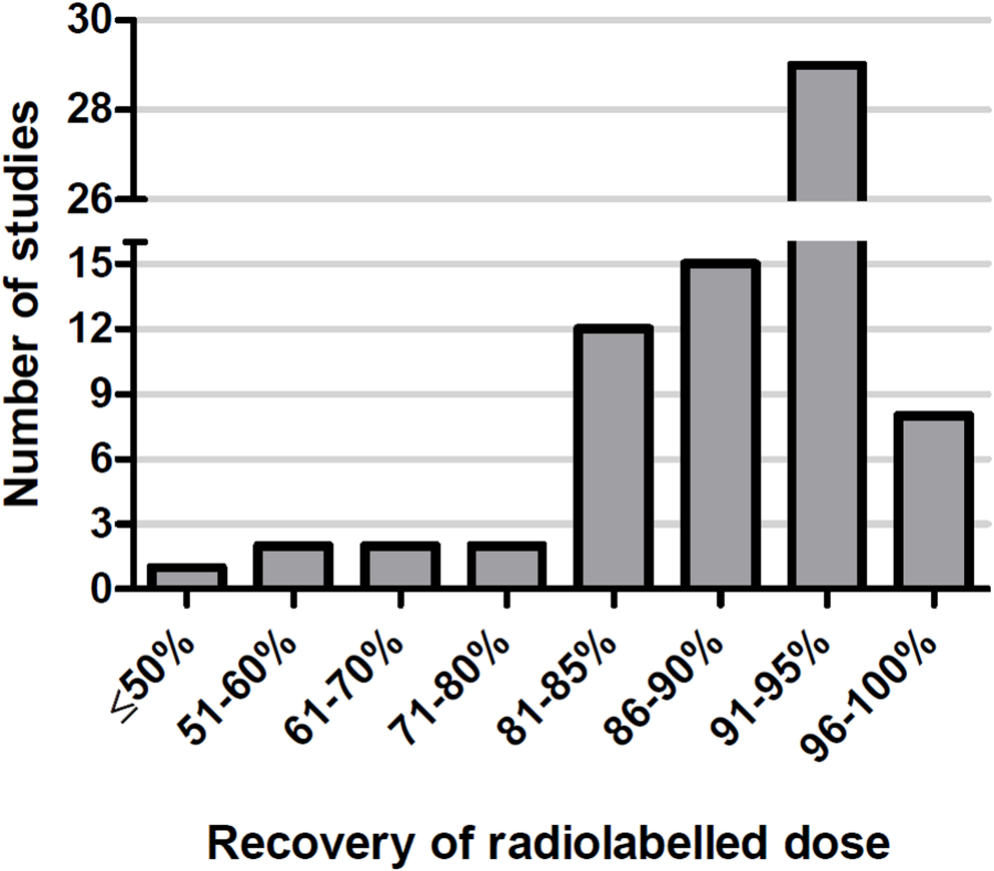
Distribution of reported radioactive dose recovery across 71 PKI mass balance studies

Discrete study durations were reported for 64 studies, ranging from 6 to 84 days, with a median of 12.5 days and an interquartile range between 9 and 18 days. Mass balance study durations are generally related to the recovery of administered radioactivity, and 64 of 71 (90%) studies reported recoveries above 80%. Thirty-seven (52%) studies even reported recoveries above 90% (see Fig. 3), in line with the preferred total recovery outlined in the recent FDA guidance [10]. Also in line with this guidance, all peer-reviewed study publications for drugs with a rather low total recovery (< 75%) discussed potential causes for low recovery and/or large variability.

Nearly all PKIs were primarily cleared hepatically, with only 6 (i.e., encorafenib, fruquitinib, ibrutinib, lorlatinib, pirtobrutinib, ruxolitinib) showing predominant recovery of total radioactivity in urine. Of the latter, encorafenib and lorlatinib featured rather equal balances (within 10% points) of recovered radioactivity between feces and urine, as was also observed for capivasertib, although a slightly higher recovery in feces was observed for this PKI.

### Pharmacogenetic considerations

Of the 48 PKIs for which mass balance study publications could be retrieved, information on the impact of genetic variation on drug PK was only found in the Materials and Methods sections of 4 studies (8%). Specifically, for imatinib, ibrutinib, and erdafitinib, *CYP2D6* metabolizer status was considered during participant inclusion. For acalabrutinib, genetic analysis was performed for selected variants in *CYP3A5* and glutathione S-transferase *GSTM1*, based on the established role of *CYP3A* and *GSTM1/2* in acalabrutinib’s metabolism. More generally, *CYP3A4* was reported as the primary/predominant drug-metabolizing enzyme for at least 59 (≥ 87%) of the studied PKIs, based on the separate EPAR analysis conducted for all PKIs. *CYP3A4* was also the predominant drug-metabolizing enzyme for imatinib, ibrutinib, and erdafitinib, for which the *CYP2D6* metabolizer status was considered during participant inclusion (see above). In this regard, imatinib’s EPAR reports that imatinib exposure “…*was not significantly increased*,* thus confirming the in vitro findings*,* i.e. CYP2D6 is not the main P450 enzyme involved…*”, while ibrutinib’s EPAR reports that “…*CYP2D6 in the metabolism of ibrutinib appears to be minimal*” and that “…*no precautions are necessary in patients with different CYP2D6 genotypes*”. In the case of erdafitinib, its peer-reviewed study publication reported that “*[w]hilst a preliminary reaction phenotyping assessment available at the time of the design of the mass balance study in humans indicated that CYP2D6 might have been involved in the erdafitinib metabolism*,* the definitive in vitro enzyme identification studies showed that CYP2C9 and CYP3A4 (but not CYP2D6) were the main enzymes*…”. Lastly, alignment with recent FDA guidance on mass balance studies was difficult to assess, as the guidance broadly recommends consideration of polymorphisms in genes coding for drug-metabolizing enzymes or transporters when determining the number of participants for enrolment [10], and sample size determination was generally not discussed in the included publications.

## Discussion

A key finding of this study is that study populations of mass balance studies performed for PKIs are seemingly homogeneous. Specifically, most of the studies were conducted in healthy, male volunteers from Western countries and consisted of rather small cohorts, including less than 10 participants. The results of our study are thereby in line with the study performed by Ramamoorthy et al. [7], although that study included a different set of drugs and only considered drugs approved during a restricted time period (from 2014 to 2018). Nevertheless, the designs observed in our study are broadly consistent with the key elements of recent FDA guidance on mass balance studies [10], despite the guidance being formalized after the included studies were conducted. In particular, 75% of studies included six or more participants, and female healthy volunteers were reported to be restricted to women of non-childbearing potential in 3 of 4 applicable cases. The inclusion of patients rather than healthy volunteers was furthermore substantiated in the case of 3 out of 7 studies that enrolled patients, with three additional studies describing roll-over or extension phases primarily for continued treatment but also for safety assessment. Additionally, multiple dose regimens were used in two studies and were supported by clear pharmacokinetic or safety-related rationales. Finally, 52% of studies reported total radioactivity recoveries above 90%, and studies with rather low recoveries all discussed potential causes of low recovery and/or large variability.

Another key observation of our study is the fact that multiple dose regimens have been used for some PKIs, namely gilteritinib and vemurafenib (which were both studied in patient populations). With such a design, the real-world use of PKIs is reflected more accurately, as also noted for gilteritinib, where marked accumulation after multiple dosing was reported (“…*reflecting its intended use*…”). Similar accumulation was described for vemurafenib that is furthermore consistent with the recent FDA guidance, which supports multiple dose mass balance studies in cases of time-dependent pharmacokinetics or when studies are conducted in patients [10]. In this context, it is unclear whether similar considerations were applied in the remaining five patient-based studies or for PKIs with autoinduction potential, including dabrafenib and encorafenib, both of which are described in their EPARs as *CYP3A4* autoinducers with steady-state being reached after 14 and 15 days, respectively.

The overall high recoveries of total radioactivity represent another key observation of our study, with most studies recovering over 80% of the administered radioactivity. This result suggests that the durations of mass balance studies were in general sufficient. Additionally, it suggests that large-scale covalent binding of the PKIs or their metabolites to (off-target) receptors is unlikely, although this cannot be excluded. It is, however, uncertain whether the pharmacokinetic parameters observed in these small-scale mass balance studies can be generalized to the actual drug users.

It is furthermore noteworthy that seven studies included patients rather than healthy participants, with three providing rationales (all related to safety considerations). In studies with patients, inclusion of female participants may hypothetically be more straightforward compared to pharmacokinetic studies in healthy volunteers, as could be derived from the fact that four of these seven studies included both males and females. In fact, these four studies (on dabrafenib, gilteritinib, lenvatinib, and vemurafenib) represent half of the studies that included females in our analysis. Interestingly, these PKIs were not developed for diseases with a clear female overrepresentation. The latter also does not seem to apply to acalabrutinib and midostaurin but would be more applicable to lapatinib and tucatinib, which represent the other four PKIs for which females were included in the mass balance studies. In this regard, it is worth noting that several other PKIs, such as abemaciclib (approved in 2018), neratinib (approved in 2018), and palbociclib (approved in 2016), are used in breast cancer treatment, as well as lapatinib and tucatinib. It remains uncertain, however, whether female (and patient) inclusion has been considered for these PKIs.

Additionally, it remains uncertain whether (PK-type) pharmacogenetic factors were broadly studied in PKI mass balance studies. Except for imatinib (approved in 2001), ibrutinib (2014), acalabrutinib (2020), and erdafitinib (2024), the retrieved publications did not report genetically predicted metabolizer or transporter statuses, as either an inclusion or exclusion criterion or in sample size determination. Admittedly, PKIs that did not have published mass balance studies were not assessed in our study due to the limited information available in the EPARs we reviewed. Still, the EPAR for one product, lazertinib (approved in 2025), did mention the inclusion of “*4 GSTM1 null and 4 non-null subjects*”, which suggests that our analysis may underestimate the extent of pharmacogenetic considerations in PKI mass balance study designs. Moreover, consideration of pharmacogenetics in mass balance studies is likely to increase in the future, due to the 2024 FDA guideline on mass balance studies which advocates the consideration of pharmacogenomics during participant inclusion [10]. Still, it should be noted that most PKIs are reported to be primarily/predominantly metabolized by *CYP3A* enzymes, for which some but rather limited pharmacogenetic recommendations are proposed from within the pharmacogenetics field [11]. For the antipsychotic drug quetiapine, for example, the Dutch Pharmacogenetics Working Group recommends an alternative drug or a dose reduction for *CYP3A4* poor metabolizers [12]. Additionally, *CYP3A*-guided pharmacogenetic dosing recommendations, with emphasis on *CYP3A5*, are available for over a decade within the transplantation field [13]. Recent work has furthermore emphasized the need to bridge an “*ethnicity gap*”, as pharmacogenomic evidence has largely focused on the CYP3A5*3 loss-of-function allele, which is more prevalent in individuals of European descent [14]. The actual relevance of pharmacogenetic testing should, however, be assessed on a case-by-case basis, as illustrated by the pharmacogenetic drug clopidogrel for which testing of a minor metabolic pathway (leading to the active metabolite, which was unknown at the time of marketing authorization [15]) is considered clinically useful [16].

A key strength of this study is that it provides a structured overview of participant inclusion in mass balance studies, covering all PKIs currently authorized by the EMA for human use and spanning the past 25 years of marketing approvals. However, by this selection procedure, withdrawn or rejected products and drugs still under development were not considered. As a result, our overview does not present a complete picture of in vivo human PKI metabolite profiling research and should be interpreted prudently. Moreover, our analysis is inherently limited by the level of detail reported in regulatory documents and peer-reviewed study publications. It is furthermore uncertain whether greater transparency in these aspects will be achieved in the future, despite recent FDA guidance on mass balance studies.

In conclusion, this study provides an overview of characteristics of participant inclusion in PKI mass balance studies, and it confirmed the expectation that these studies are generally rather homogeneous, typically being conducted in small cohorts of around 6 participants. Furthermore, these studies were primarily performed in healthy male volunteers from Western countries and usually employed a single-dose regimen. Additionally, genetically predicted metabolizer statuses were rarely mentioned in the context of participant inclusion, yet this may change in the near future due to specific pharmacogenetic considerations outlined in a recently established regulatory guidance on mass balance studies.

## Supplementary Information

Below is the link to the electronic supplementary material.

Supplementary Material 1

## Data Availability

All data supporting the findings of this study are available within the paper and its Supplementary Information.
